# Work empowerment among cancer care professionals: a cross-sectional study

**DOI:** 10.1186/s12913-021-06528-8

**Published:** 2021-05-25

**Authors:** Mervi Siekkinen, Liisa Kuokkanen, Hannele Kuusisto, Helena Leino-Kilpi, Päivi Rautava, Maijastiina Rekunen, Laura Seppänen, Minna Stolt, Leena Walta, Virpi Sulosaari

**Affiliations:** 1 Turku University Hospital, FICAN West Cancer Centre, P.O. Box 52, FI-20521 Turku, Finland; 2 Helsinki University Hospital, Helsinki, Finland; 3 Turku University of Applied Sciences, Turku, Finland; 4 University of Turku, Department of Nursing Science, Turku University Hospital, Turku, Finland; 5 University of Turku, Public Health and Turku University Hospital, Clinical Research Services, Turku, Finland; 6 Finnish Institute of Occupational Health, Helsinki, Finland

**Keywords:** Empowerment, Work, Cancer care, Personnel, Interprofessional

## Abstract

**Background:**

There is a growing understanding that empowerment of interprofessional personnel is linked to job satisfaction levels and quality of care, but little is known about empowerment in the context of cancer care. This study describes how interprofessional cancer care personnel perceive their performance and factors that promote work empowerment.

**Methods:**

This cross-sectional study enrolled 475 (45.2%) of the 1050 employees who work at a regional cancer centre. The participants used two self-administered questionnaires – the Performance of an Empowered Personnel (PEN) questionnaire and Work Empowerment Promoting Factors (WEP) questionnaire – to report perceptions of work empowerment. Both questionnaires’ categories comprise moral principles, personal integrity, expertise, future orientation, and sociality. The data were analyzed using IBM SPSS Statistics, Versions 24 and 25.

**Results:**

Overall, the performance of work empowerment was evaluated as being rather high (overall sum score mean: 4.05; range: 3.51–4.41; scale: 1–5). The category that rated highest was moral principles (4.41), and the one rated lowest was the social category (3.51). The factors that promoted work empowerment also ranked high (3.93; range: 3.55–4.08; scale: 1–5), with personal integrity (4.08) the highest and future orientation (3.55) the lowest. Performance and factors that promoted work empowerment correlated positively, moderately, and highly statistically significantly (r = 0.531; *p* < 0.001). Statistically significant associations also were found between empowered performance of personnel and empowerment promoting factors (sex, education, leadership position, belonging to an interprofessional team, and time elapsed since training in interprofessional cooperation).

**Conclusion:**

The personnel rated their performance and the factors perceived to promote work empowerment rather highly. Personal empowerment can be promoted through teamwork training and supportive management in interprofessional cancer care.

## Background

Cancer is one of the most common diseases in developed societies globally. In 2018, 3 million new cases of cancer are expected to be diagnosed in the 28 EU member states [1]. In Finland, the annual number of patients diagnosed with cancer has been increasing steadily, and in 2030, approximately 43,000 new cancer cases are expected to be diagnosed in the country, which currently has a population of 5.5 million, 11,000 more than in 2013. This marked increase is due to the demographic effects from an increasing elderly population, among whom the incidence of cancer is the highest [2].

Cancer care in Finland is provided through five Cancer Centre regions that the National Cancer Centre Finland (FICAN) coordinates to harmonize, improve, and streamline cancer treatment nationwide, with the ultimate goal of providing all cancer patients with standardized, high-quality cancer care. Each regional Cancer Centre is, in itself, a network of interprofessional health care workers and scientists centreed around one of the five university hospitals, with clinical units in the central and other hospitals in each region.

Cancer care requires effective collaboration by an interprofessional health care team. *Interprofessional collaboration* is defined as collaborative interaction among experts with different professional backgrounds involved – in this case, to care for cancer patients – but with common goals [3–6]. The present study’s coordinating cancer centre (FICAN West Cancer Centre) was became as a member of the Organization of Cancer Institutes (OECI) in 2019 by the OECI Accreditation and Designation Program [7]. According to the first audit report from the OECI in 2017, the FICAN West Cancer Centre had shortcomings related to interprofessional collaboration, creating a strong challenge for the FICAN West Cancer Centre to understand the phenomenon better, resulting in the present study.

The patient is at the core of collaboration. Cancer patients’ care pathways are unique for each individual, and care might involve anything from surgery to radiotherapy, chemotherapy, and other cancer treatments at one or two hospitals with several appointments. This situation has led to personnel working not only within the hospital where they are employed, but also in hospital networks with personnel from different cancer care wards, in outpatient departments, and in departments providing supportive care. Professional expert work is patient-driven, collaborative, and team-based, and these personnel work together to deliver the highest quality of care with shared objectives, decision-making, and other responsibilities. Collaboration is usually interactive, and multidisciplinary teams communicate with each other to manage individual patients’ cases and agree on care plans [8–10]. The team caring for cancer patients may comprise a nurse, surgeon, oncologist, radiographer, physicist, physiotherapist, social worker and/or other specialists, depending on need.

In an earlier study on interprofessional cancer care, we concluded that systematic and continuous evaluation is needed if a work community is to develop and improve [11]. Other studies have demonstrated that collaboration and integration among professionals from different disciplines may be challenging and that disciplinary boundaries may hinder interprofessional collaboration [8]. The personnel in different work situations adopt different, and sometimes conflicting, clinical approaches; physicians and nurses construct discipline-specific professional identities; and conflicts emerge between professionals from different disciplines [12]. Typically, the physician participates in the decision-making process related to the medical treatment of cancer patients, while the nurse knows best how to implement and supplement treatment, as well as promote treatment success, e.g., by collaborating with a physiotherapist or clinical dietitian. The experience of poor care quality is associated with too little interprofessional teamwork [10], and a lack of cooperation among professionals prevents work empowerment [13].

### Work empowerment

The term *empowerment* has been used to describe the essence of human existence and development, a process in which an individual feels integrated and confident in his or her personal abilities, while defending personal rights in his or her own life. It also denotes aspects of organizational effectiveness and quality. The empowerment ideology is rooted in social action, in which empowerment is associated with community interests and attempts to increase the power and influence of oppressed groups (such as workers, women, and ethnic minorities) [14].

Work empowerment refers to an individual having the experience of carrying out tasks related to his or her work. Empowerment is an umbrella concept for work-related professional growth and development [15]. Work produces pleasure and allows workers to experience power. An empowered caregiver relies on his or her own professional skills and abilities, and wants to implement and develop these skills and abilities. Empowered personnel value their own skills and perceive their work as meaningful. The development of oneself and the work community provides a resource, as well as a goal. Empowerment enables autonomy at work, and the way to increase empowerment is to allow autonomy. However, it must be recognized that empowerment is not a permanent state and that empowerment levels vary over time [16].

This study presents the theoretical framework of work empowerment developed by Kuokkanen and Leino-Kilpi 2001 [13]. The framework emerged from theme-based interviews on empowerment [13] and the psychological model of empowerment by Thomas and Velthouse 1990 [14, 17]. The individual’s characteristics and actions at work turned out to be important for the model. The Model of Nurse Empowerment described what an empowered nurse is like (Qualities of an Empowered Nurse, QEN) and how s/he performs her/his tasks (Performance of an Empowered Nurse, PEN, in this study adapted to Empowered Personnel) in relation to the factors that promote empowerment (Work-related Empowerment Promoting factor, WEP) and impede empowerment (Work-related Empowerment Inhibiting factor, WEI) (14). Five categories were formed based on these factors: moral principles; personal integrity; expertise; future-orientedness; and sociability [13, 18]. For the present study, PEN and WEP, were adopted as the conceptual framework for the present study’s questionnaire with two changes in the original variables of the questionnaires (Table 1).

**Table 1:** The performance of an Empowered Nurse (PEN) and Work Empowerment Promoting factors (WEP) ^(13,18)^

Work Empowerment Promoting factors (WEP)	Performance of an Empowered Nurse (PEN)
**Moral principles**	**Moral Principles**
Shared values	Treats other with respect
Esteem for others	Act honestly
Concerted care philosophy	Acts justly
**Personal integrity**	**Personal integrity**
Delegated responsibilities	Looks after own well-being
Confidence	Dares to say and act
Feedback	Acts effectively under pressure Acts flexibly
**Expertise**	**Expertise**
Evaluation and development	Acts skilfully
**Cooperation** (between nurses and between different professionals)^b^	Makes decisions ***Act independently***^a^
Training	Consults and teaches colleagues
**Future-orientedness**	**Future-orientedness**
Continuity of work	Finds creative solutions
Position opportunities	Promotes new ideas at work
Access to information	Acts after planning, assesses effects
**Sociability**	**Sociability**
Collegial support	Discusses openly
Problem solving	Works for the common goal
Open ambience	Solves problems

^a^In this study for the questionnaire adapted with permission from original developer from acting independent in nursing to acting independent in care, ^b^ adapted from cooperation between nurses to cooperation between my own profession

Earlier studies have demonstrated that work empowerment is perceived as being positive and highly valued within health care. These studies demonstrated that personnel feel the most empowered in the moral principles domain and the least empowered in the sociality domain. Age, long work experience, and higher education levels increase the sense of empowerment, which is related to job satisfaction, remaining in the same field or discipline, job stress, and advancement [13, 19–21].

A study among interprofessional teams rated work empowerment quite high [22]. Of the promoting factors, the category of future-orientedness scored the lowest (continuity of work, opportunities for advancement, and access to information). Of the impeding factors, the same category of future-orientedness scored the highest (organizational bureaucracy and hierarchy, authoritarian leadership, poor access to information, and brief temporary positions) [22]. At times of organizational changes, the factors that promoted work empowerment were job control, possibilities for developing working practices, and organizational equality [23].

Although work empowerment has a strong position in cancer care organizations, the literature on work empowerment in this setting is limited. Thus, we have studied work empowerment in the context of cancer care.

Our research questions were:

1. How do interprofessional cancer care personnel characterize their performance of work empowerment?
2. How do interprofessional cancer care personnel characterize the factors that promote work empowerment?
3. What is the relationship between performance and the factors that promote work empowerment among cancer care professionals?

## Methods

### Study design and participants

The data for this cross-sectional study originated from a research project, the VETÄVÄ project, which was designed to enhance empowerment and interprofessional collaboration among health care personnel involved in cancer care [24].

We randomly recruited cancer care personnel working at one Cancer Centre region, the FICAN West Cancer Centre (Table 2. Sample characteristics). This Cancer Centre comprises one university hospital and two central hospitals. The data set (total *n* = 475, 45.2%) was collected between May and October 2018 (response rate: 33.3%) and between November and December 2019 (response rate: 11.9%), following the research plan from the VETÄVÄ project (first data collection: 2018; second data collection: 2019). An electronic information letter about the study and its purpose, which included a link to the online survey, was sent to all healthcare professionals working with patients in the FICAN West Cancer Centre. Out of the 1050 professionals to whom the letter was sent, 490 (47%) opened the online survey. Considering that professionals in some units had technical problems with the online survey, the option to fill out the survey on paper was provided (*n* = 80). Altogether, 29 of all returned surveys were excluded due to a lack of substantial data, not meeting inclusion criteria (i.e., not treating cancer patients), and double submissions.

**Table 2:** Sample characteristics

				n	%	
**Sex**				429		
Female				357	83.2	
Male				72	16.8	
**Work experience in cancer care**				432		
Daily				281	65.0	
Weekly				91	21.1	
Monthly				40	9.3	
Less than monthly				20	4.6	
**Title**				430		
Registered nurse				226	52.6	
Medical specialist				36	8.4	
Radiographer				27	6.3	
Practical nurse				25	5.8	
Head nurse				21	4.9	
Senior physician or assistant senior physician				19	4.4	
Staff nurse				15	3.5	
Midwife				9	2.1	
Dietician				7	1.6	
Other professions				29	10.5	
**Time passed since training of interprofessional co-operation**				332		
Last year				176	53.0	
Last 2–3 years				80	24.1	
Last 5 years				29	8.7	
Over 5 years ago				47	14.2	
**Leadership position**				435		
Yes				67	15.8	
No				358	84.2	
**Numerical background variables**				**N**	**mean ± sd** **(min-max)**	
**Age**				338	43.6 ± 11.9 (22–67)	
**Work experience in healthcare**				350	17.4 ± 11.9 (0–44)	
**Work experience in cancer care**				350	13.2 ± 10.7 (0–41)	
		**mean**	**sd**	**min**	**max**	**n**
**K2**	Age	43.6	11.9	22	67	338
**K8**	Work experience in healthcare	17.4	11.9	0	44	350
**K9**	Work experience in cancer care	13.2	10.7	0	41	350

### Questionnaires

The questionnaires were administered in Finnish, the recipients’ vernacular.

The Performance of an Empowered Personnel (PEN) and Work Empowerment Promoting Factors (WEP) questionnaires were used to collect the data on work empowerment, and these questionnaires were developed and validated for this purpose (18, 25) based on the model of psychological work empowerment developed on the foundation of Thomas and Velthouse’s (1990) motivation theory [14, 17]. The data extracted from the questionnaires were used to describe the level of each respondent’s empowerment in different nursing environments [23, 24] and for multi-professional personnel [23]. To capture the phenomenon in the context of interprofessional cancer care and follow a theoretical review format [16], the word *nursing* was changed to the word *care* in two instances in the WEP questionnaire: 1) “I work independently in nursing –> I work independently at work” and 2) “My work unit/organization has collaboration between nurses –> My work unit/organization has cooperation within my own professional group.” The PEN (19 items) and WEP (18 items) are divided into five categories (range: 1–5, 1 = totally disagree and 5 = totally agree), with high average values in the category scores indicating high empowerment. The categories in both questionnaires are moral principles, personal integrity, expertise, future orientation, and sociability.

In the PEN questionnaire, “moral principles” reflect human values in nursing (treats others with respect, acts honestly, acts justly). “Personal integrity” refers to mastery of one’s own life and also is expressed by recognizing one’s own resources (looks after one’s own well-being, dares to speak out and act, acts effectively under pressure, acts flexibly). “Expertise” manifests itself as professional competence and as having a wide range of knowledge (acts skillfully, makes decisions, acts independently, consults and teaches colleagues). “Future-orientedness” involves creativity and innovation (finds creative solutions, promotes new ideas at work, acts after planning, assesses effects). “Sociability” refers to socially skilled personnel who are flexible and can create a positive ambience in the workplace (discusses issues openly, works for common goals, solves problems).

In the WEP questionnaire, “moral principles” reflect human values in nursing (shared values, esteem for others, concerted care philosophy). “Personal integrity” refers to mastery of one’s own life and also is expressed by recognizing one’s own resources (delegated responsibilities, confidence, feedback). “Expertise” manifests itself as professional competence and as having a wide range of knowledge (evaluation and development, cooperation, training). “Future-orientedness” involves creativity and innovation (continuity of work, position opportunities, access to information). “Sociability” refers to socially skilled personnel who are flexible and can create a positive ambience in the workplace (collegial support, problem solving, open ambience).

Both questionnaires’ content validity, criterion-related validity, and construct validity have been tested in earlier studies [18, 23, 25, 26]. The content, criterion-related, and construct validities have been tested in an earlier study by Kuokkanen et al. [18]. The reliability alpha coefficient has varied between 0.87 and 0.88 (PEN) and between 0.89 and 0.92 (WEP) [22, 23, 26, 27]. For this sample, the Cronbach’s alpha coefficient for the QEN subcategories ranged from 0.59 to 0.85.

### Other variables

In addition to demographic data, background data comprised age, sex (man, woman, other), work experience in general care and cancer care (in years), professional title (registered nurse, medical specialist, radiographer, practical nurse, head nurse, senior physician or assistant senior physician, staff nurse, midwife, dietitian, or other professional), memberships on interprofessional teams (yes, no), time elapsed since training on interprofessional cooperation (1 year or less, two to 3 years, less than 5 years, over 5 years), and leadership position (yes, no).

### Data analysis

The data were analyzed statistically using IBM SPSS Statistics, Versions 24 and 25 (IBM Corp.). Frequency tables and descriptive statistics were generated, and sum variables were structured according to the WEP and PEN questionnaires’ subcategories (moral principles, personal integrity, expertise, future orientation, and sociability) [13]. The mean was formed by adding up the values ​​of the questions and dividing by the number of values. Missing data were coded blank. The Cronbach’s alpha coefficient was used to check the sum variables’ internal consistency. Associations between performance level, promoting factors, and two-category background variables were tested using the Mann-Whitney U-test. Associations between numerical variables were described using Spearman’s (r_s_) and Pearson’s (r) correlation coefficients. Generalized linear models were used to identify background factors independently associated with work empowerment and promoting factors. Statistical significance was set at < 0.05.

### Ethics

Good scientific practice was followed throughout the study [28]. Ethical approval (Ethics board of the University of Turku, Finland: statement 48/2017) and organizational permission from each participating organization were obtained. Information about the study and its purpose was given in writing to all potential participants. Voluntary, anonymous, and confidential participation was emphasized. All invitees had the opportunity to decline participation without giving any reason. A completed survey was viewed as the respondent’s consent to participate. The immaterial rights to the use of the PEN and WEP questionnaires are owned by Dr. Liisa Kuokkanen, PhD, who gave permission to use and modify the questionnaires.

## Results

### Sample characteristics

The participants’ mean age was 43.4 (range: 22–67, SD = 11.8) (Table 2).

Most respondents were female (*n* = 357, 83.2%), had participated in the daily care of cancer patients (*n* = 281, 65%), and had more than 5 years of experience in cancer care (*n* = 306, 70.7%; mean: 13.0 years). Half of the respondents (*n* = 227, 54.2%) were working in surgical care, and a third (*n* = 158, 37.7%) in oncology. Most respondents did not have managerial positions (*n* = 358, 82.3%) and were part of some interprofessional team (*n* = 307, 75.1%) (Table 2).

Of the respondents, 76.8% had a nursing degree (*n* = 334), e.g., nurse, radiographer, midwife; 15.2% had a medical degree (*n* = 66), e.g., physicists and dentists; and 8% had some other healthcare degree (n = 35), e.g., physiotherapist, rehabilitation professional, or other professional, such as dietitian, social worker, pastor, or physicist. Most (*n* = 332, 76.3%) had participated in complementary education related to interprofessional collaboration. (Table 2).

### Performance of work empowerment

The personnel ranked their performance level on work empowerment rather high, with a mean of 4.05 (scale: 1–5; 1 = totally disagree and 5 = totally agree). The work empowerment level subcategories ranged from 4.41 to 3.51 (Table 3).

**Table 3:** The Cronbach’s alpha coefficients of PEN and WEP sum variables in cancer care

		PEN			WEP		
		mean^a^	α	N	mean^b^	α	N
All categories		4.05	0.863	406	3.93	0.926	407
	Moral principles	4.41	0.630	426	3.96	0.793	428
	Personal integrity	4.15	0.604	429	4.08	0.655	430
	Expertise	4.19	0.661	425	4.06	0.777	421
	Future orientation	3.85	0.771	423	3.55	0.846	424
	Sociability	3.51	0.593	424	4.01	0.753	425

^a^ = scale 1 = not at all confident, …, 5 = fully confident

^b^ = scale 1 = totally disagree, …, 5 = totally agree

*PEN* Performance of an Empowered Personnel

*WEP* Work Empowerment Promoting Factors

The performance of work empowerment focused on the moral principles category (4.41). The respondents assessed the item, “I treat all people with respect in my work, regardless of circumstances,” with the highest (mean: 4.75; SD 0.46). The lowest-assessed category was sociability (3.51), and the lowest-rated item was “I work in special positions/duties concerning the entire work community or organization” (mean: 2.63; SD: 1.57) (Table 3).

The five categories’ internal consistency was evaluated using Cronbach’s alpha coefficients and ranged from 0.593 to 0.771 (Table 3).

### Factors promoting work empowerment

The personnel ranked the promoting factors rather high, with the total mean of all categories at 3.93 (scale: 1–5; 1 = totally disagree and 5 = totally agree). The five empowerment categories’ means ranged from 4.08 to 3.55 (Table 3). Of the empowerment-promoting factors, the lowest-assessed was the personal integrity category (4.08). The highest-rated item was “I get help from my colleagues when I need it” (mean: 4.61; SD: 0.64). The category that was assessed the lowest was future orientation (3.55), and the item rated the lowest was “I get enough information about the goals and results of the work unit and the organization” (mean: 3.38; SD: 1.13) (Table 3).

The five categories’ internal consistency was evaluated using the Cronbach’s alpha coefficient, which ranged from 0.655 to 0.846 (Table 3).

### Relationship between performance and factors promoting work empowerment

Those who experienced higher performance of work empowerment reported more promoting factors. The performance and promoting factors of work empowerment correlated positively, moderately, and highly statistically significantly (r = 0.531, *p* < 0.001).

### Relationship between performance and factors promoting work empowerment and background variables

Several factors were associated with work empowerment – some with performance only, some with promoting factors only, and some with both. There were statistically significant relationships between performance and promoting factors, and background variables, such as age, sex, work experience, work experience in cancer care, management position, education degree, belonging to an interprofessional team, and time elapsed since interprofessional cooperation training (Table 4).

**Table 4:** Relationships between PEN and WEP and background variables

Variables	PEN	N	***p***-value	WEP	N	p-value
Age	r = 0.109	414	0.026	ns^1^		
Work experience in health care	r = 0.156	431	0.001	ns^1^		
Work experience in cancer care	r = 0.171	429	< 0.001	ns^1^		
Sex	mean^2^		0.001^3^	mean^4^		< 0.001^3^
Male	4.22	70		4.23	69	
Female	4.02	355		3.87	425	
Leadership position	mean^2^		< 0.001^3^	mean^4^		< 0.001^3^
Yes	4.32	67		4.30	66	
No	4.01	354		3.86	355	
Education status	mean^2^		< 0.001^3^	mean^4^		0.002^3^
Nursing care	4.02	331		3.89	66	
Doctor	4.28	66		4.12	331	
Belonging to interprofessional team	mean^2^		< 0.001^3^	ns^1^		
Yes	4.21	102				
No	4.00	303				
Time passed since training of interprofessional co-operation	ns^1^			mean^4^		0.001^3^
Maximum 5 years				4.00	284	
Over 5 years				3.59	46	

^1^ = not significant

^2^ = scale 1 = not at all confident, …, 5 = fully confident

^3^ = Mann Whitney U-test

^4^ = scale 1 = totally disagree, …, 5 = totally agree

*PEN* Performance of an Empowered Personnel

*WEP* Work Empowerment Promoting Factors

#### Relationship between performance and background variables

The respondents’ ages correlated very weakly, positively, and statistically almost significantly with performance of work empowerment. The older the respondent, the higher the performance (r = 0.109, *p* = 0.026). The same relationship emerged when the different age groups’ means were compared (Table 4). Empowerment was categorized lower among the respondents who were below age 30 and almost was significantly lower among participants who were below age 30 compared with those age 55 and up (*p* = 0.013), and very significantly lower compared with those ages 30–54 (*p* = 0.001; Kruskal-Wallis test, Bonferroni’s correction) (Table 4).

Work experience also correlated with performance of work empowerment. Work experience in health care (r = 0.156; p = 0.001) and especially in cancer treatment (r = 0.171; *p* < 0.001) correlated weakly, positively, and statistically very significantly with performance (Table 4).

There was an association between work experience in cancer care and performance of work empowerment in the group of respondents who had worked in cancer care less than or more than 5 years: The respondents who had more than 5 years of work experience reported a higher level of empowerment than those who had less work experience (means: 3.93 vs. 4.11; *p* < 0.001; Mann-Whitney U-test) (Table 4).

Personnel on the interprofessional team were more empowered than others, as belonging to a multidisciplinary team was statistically very significantly associated with performance (means: 4.21 vs. 4.00; p < 0.001, Mann-Whitney U-test), but not with the experience of promoting factors (*p* = 0.138, Mann-Whitney U-test) (Table 4).

#### Relationship between promoting factors and background variables

The time since the previous episode of complementary training in the field of interprofessional cooperation correlated weakly, negatively, and almost statistically significantly with the respondents’ experience of factors promoting work empowerment. The less time that had passed since training, the higher the level of feeling empowered. (r_s_ = − 0.116; *p* = 0.035; Spearman). There was also a statistically significant difference between the group that had participated in training less than 5 years or more than 5 years previously (means: 4.00 vs. 3.59; *p* = 0.001, Mann-Whitney U-test) (Table 4).

#### Relationship between performance and promoting factors and background variables

There was a difference between sexs: Male respondents perceived more performance of work empowerment and also more promoting factors than female respondents (means: 4.22 vs. 4.02; p = 0.001 and 4.23 vs. 3.87; *p* < 0.001, respectively, Mann-Whitney U-test) (Table 4).

Physicians reported more performance of work empowerment compared with nursing personnel (nurses, radiographers, and midwives) working in cancer care (means: 4.28 vs. 4.02; p < 0.001, Mann-Whitney U-test) and significantly more promoting factors (means: 4.12 vs. 3.89; *p* = 0.002, Mann-Whitney U-test).

A managerial position increased the experience of work empowerment, as supervisors had higher performance of work empowerment and experienced more promoting factors than non-supervisors. The difference between these is statistically very significant (means: 4.32 vs. 4.01; p < 0.001 and 4.30 vs. 3.86; p < 0.001, Mann-Whitney U-test) (Table 4).

The association between the respondents’ background variables and work empowerment was modelled using generalized linear models. Having a managerial position was associated independently with performance of work empowerment (df = 1, F = 7.299, *p* = 0.007). Sex (*p* = 0.006), leadership position (*p* = 0.033), and the time elapsed since receiving complementary training in the field of interprofessional cooperation (*p* = 0.013) were associated independently with the factors promoting work empowerment (Table 5).

**Table 5:** Relationships between WEP and background variables based on generalized linear model

Background variables	df	F	p
Sex	1	7.750	0.006
Leadership position	1	4.622	0.033
Time passed since training of interprofessional co-operation	3	3.690	0.013

*WEP* Work Empowerment Promoting Factors

## Discussion

### Results compared with earlier studies

This study examined work empowerment in the context of interprofessional cancer care from the perspective of performance and promoting factors. We found that personnel self-assessed their empowerment levels quite high, and that a significant, positive correlation existed between performance and empowerment-promoting factors, corresponding with a study by Kuokkanen and Leino-Kilpi 2001 [13].

Our data suggest that moral principles largely support performance of work empowerment. Sociability was rated lowest in relation to empowerment, with the number of factors that promote empowerment the highest in the category of personal integrity and the lowest in the category of future0orientedness. Similar results have been reported in previous studies on empowerment of nurses [22, 23, 25, 29].

Moral principles are important in patient-Centreed health care. Personnel perceived that they should treat all people professionally and with respect, regardless of circumstances. Earlier studies have demonstrated that the ethics of treating human beings with respect and honesty strongly pervade work done in strictly organized healthcare organizations [29]. However, sociability is the least-empowering category (Table 3), which might be related to the lack of positions involved in the entire work community or organization. Our results indicate that increased efforts are needed to implement a culture of delegating special tasks to personnel, which would foster career development further and allow for new influences to enter the working community. Such elements are known to support empowerment [13]. Earlier studies have demonstrated that special duties may introduce a change in individuals and teams’ social performance as responsibilities change, generating a need for responses between interprofessional team members [18, 30, 31].

An interesting finding related to the empowerment-promoting factors was that personnel were most critical to future-orientedness (continuity of work, position opportunities, access to information). Specifically, the personnel reported that too little information was provided regarding the work unit and organizational goals and results. This finding corresponds with earlier studies [32]. Our results imply that information within the healthcare team, in which shift work is the norm, did not reach all personnel simultaneously. Empowerment requires that all members of the organization know and acknowledge the work objectives and are committed to organizational goals [10]. Adequate and timely information sharing, crucial for successful interprofessional cooperation [11], presents a major challenge for management. Shared information might increase feeling of competence and confidence of their knowledge and skills, which are important empowering aspects of expertise [13, 33, 34].

This study’s evidence elicits the need to examine some performance relationships, factors that promote work empowerment, and background factors more carefully. Our results support the view that male sex, age, and number of years that an individual has been working in cancer care are related to experience: Participants older than 30 and those who have worked longer in cancer care felt more empowered than younger and less-experienced participants. These findings partly confirmed some earlier studies’ results, according to which, newly graduated nurses [15], nurses with less than 5 years’ experience, and nurses with more than 30 years of work experience [24] reported fairly high empowerment levels. Our study implies that longtime expertise in cancer care increases the subjective experience of power and competence, important qualities for an empowered professional [13]. Earlier studies among cancer care professionals indicated that critical introspection and outside guidance during the work career improve integrity, self-reliance, and the skills and experience needed to master cancer care [34]. Co-workers and supervisors’ mentoring and support are important and need to be emphasized as well [35].

In the present study, positive associations were found between interprofessional work-related background variables (training and belonging to a team) and experiencing work empowerment. The less time that had elapsed between training sessions in interprofessional cooperation, the higher the number of factors that contributed to empowerment. Our study also suggests that personnel who are part of interprofessional teams feel more empowered than personnel outside of such teams. It had been known that empowerment is strengthened by providing personnel with opportunities to update their expertise [13, 23, 33], as well as participate in interprofessional meetings [26, 31, 36]. The present study demonstrates that interprofessional cooperation and work empowerment are interrelated, challenging management to support these activities through education and cultivate teamwork.

The study participants’ position in the process of cancer patient care was related to experienced work empowerment (Table 4). Care team leaders and physicians felt more empowered in their work than others, and physicians identified more work empowerment-promoting factors than nursing personnel. Earlier studies found that a managerial position promotes future-orientedness [26] and that personnel in managerial positions who have received additional education [32, 33] feel more empowered, probably because they have more theoretical knowledge than non-managers and have expert roles, qualities known to be related to empowerment [13]. In interprofessional networking, it is important to pay attention to formal, as well as informal, hierarchies [37]. The basis of empowerment – the opportunity to participate in decision making – is different for different professionals at different levels [8]. Thus, a physician decides on the medical treatment of cancer patients, and the nurse’s role is to implement and supplement the treatment through nursing and to promote successful treatment outcomes, which are related to the physician’s decisions. This contrasts with the process of co-creation, in which professionals come together in peer-like interaction to address problems jointly to develop more responsive, integrated, and outward-looking health care systems [37]. The possibility of participating in decision-making clearly needs to be supported in all positions.

It was very satisfying that interprofessional personnel experienced quite a high degree of empowerment in the present study, which involved professionals working in specialized care organized across several hospital districts under the responsibility of university hospitals. Careers in the interprofessional care setting in a cancer Centre network face challenges related to work development [34]. However, work development is an important element contributing to empowerment [22]. Earlier studies have suggested that getting to know personnel on an individual basis and sharing power and responsibility may be needed to promote a sense of empowerment [32]. Obviously, this challenges management to support individual employees’ work empowerment through actions, as well as through effective planning.

### Study limitations

This is apparently the first study on work empowerment within a Finnish cancer Centre region. This cross-sectional study included professionals in cancer patient management and support personnel from a given university hospital and two central hospitals, and the results may be generalized to other organizations to some extent. However, additional research is needed in other cancer Centre regions to examine our results’ generalizability. Bias due to the limited sample size is possible, considering that the response rate was only 45.2%. Furthermore, the response rate was not affected to any significant degree by repeated letter reminders and a video distributed to the cancer care units during data collection. However, the rates for various professionals who responded were proportionate to the entire FICAN West cancer Centre’s staff. Also, with the available data, we were able to identify relationships between background factors and factors promoting work empowerment. Our study is unique in that the participants represented all interdisciplinary personnel involved in cancer care.

## Conclusion

In this paper, we examined job performance and factors promoting empowerment and their relationship to background factors among interprofessional personnel working in cancer care. The study indicated that the variables related to empowerment in cancer care are duration of employment in the field of cancer care, degree of education, and hierarchical position. Thus, the longer the duration of working in this field, the higher the level of education, and being in a managerial position, the higher the level of empowerment. Regarding interprofessional activities, cooperation and teamwork support work empowerment experiences.

Personnel should attend continuing education courses and training to develop professionally in their fields of expertise. To promote personnel’s future-orientedness, it is important to maintain the option of a career path and guarantee access to information. Personnel also should participate in the organization’s planning and development efforts, thereby supporting their personal future-orientedness.

Further national and international research is needed to examine and follow the development of interprofessional cancer care and how the empowering process affects cancer care.

## Data Availability

The data sets analyzed in this study are available from the corresponding author to a reasonable extent.

## Abbreviations

PEN: Performance of Empowered Personnel
WEP: Work Empowerment Promoting Factors
EU: European Union
FICAN: National Cancer Centre Finland
OECI: Organization of Cancer Institutes
VETÄVÄ: Research consortium “The Future Magnetic Cancer Centre”
